# Risk factors for increased left ventricular hypertrophy in patients with chronic kidney disease: findings from the CKD-JAC study

**DOI:** 10.1007/s10157-018-1605-z

**Published:** 2018-06-27

**Authors:** Kosaku Nitta, Satoshi Iimuro, Enyu Imai, Seiichi Matsuo, Hirofumi Makino, Tadao Akizawa, Tsuyoshi Watanabe, Yasuo Ohashi, Akira Hishida

**Affiliations:** 1 Department of Medicine, Kidney Center, Tokyo Women’s Medical University, Tokyo, 162-8666 Japan; 2 Teikyo Academic Research Center, Teikyo University, Tokyo, Japan; 3 Nakayamadera Imai Clinic, Hyogo, Japan; 4 Department of Nephrology, Nagoya University, Aichi, Japan; 5 Okayama University, Okayama, Japan; 6 Division of Nephrology, Department of Medicine, Showa University School of Medicine, Tokyo, Japan; 7 Japan Organization of Occupational Health and Safety Fukushima Rosai Hospital, Fukushima, Japan; 8 Department of Integrated Science and Engineering for Sustainable Society, Chuo University, Tokyo, Japan; 9 Yaizu City Hospital, Shizuoka, Japan

**Keywords:** Chronic kidney disease, Left ventricular hypertrophy, Hypertension, Body mass index, Albuminuria, Mineral metabolism, Antihypertensive agent

## Abstract

**Background:**

Although left ventricular hypertrophy (LVH) has been established as a predictor of cardiovascular events in chronic kidney disease (CKD), the relationship between the prevalence of LVH and CKD stage during the pre-dialysis period has not been fully examined.

**Methods:**

We measured left ventricular mass index (LVMI) in a cross-sectional cohort of participants in the Chronic Kidney Disease Japan Cohort (CKD-JAC) study to identify factors that are associated with increased LVMI in patients with stage 3–5 CKD.

**Results:**

We analyzed the baseline characteristics in 1088 participants (male 63.8%, female 36.2%). Diabetes mellitus was the underlying disease in 41.7% of the patients, and mean age was 61.8 ± 11.1 years. LVH was detected in 23.4% of the patients at baseline. By multivariate logistic analysis, independent risk factors for LVH were past history of cardiovascular disease [odds ratio (OR) 2.364; 95% confidence interval ([CI) 1.463–3.822; *P* = 0.0004], body mass index (OR 1.108; 95% CI 1.046–1.173; *P* = 0.0005), systolic blood pressure (OR 1.173; 95% CI 1.005–1.369; *P* = 0.0433), urinary albumin (OR 1.425; 95% CI 1.028–1.974; *P* = 0.0333), and serum total cholesterol level (OR 0.994; 95% CI 0.989–0.999; *P* = 0.0174).

**Conclusion:**

The cross-sectional baseline data from the CKD-JAC study shed light on the association between LVH and risk factors in patients with decreased renal function. Further longitudinal analyses of the CKD-JAC cohort are needed to evaluate the prognostic value of LVH in CKD patients.

## Introduction

Chronic kidney disease (CKD) is the leading risk factor for cardiovascular disease (CVD), a great threat to health and an economic burden [1]. In Japan, the prevalence of end-stage kidney disease (ESKD) requiring renal replacement therapy has been increasing over the last three decades. There were 38,327 new cases in 2014, bringing the total number of cases in Japan to 320,448 [2]. Since the number of patients requiring dialysis has continued to increase [3], there appears to be an enormous number of latent cases of CKD in the Japanese population. In a recent study, Imai et al. reported the prevalence of CKD by calculating the estimated glomerular filtration rate (eGFR) using an equation that estimates GFR based on data from the Japanese annual health check programing 2005 [4]. They predicted that 13% of the Japanese adult population (approximately 13.3 million people) would have CKD in 2005. Mortality, predominantly due to cardiovascular events, is high in patients with CKD and left ventricular hypertrophy (LVH) is a strong risk factor [5].

Renal dysfunction and albuminuria in CKD patients have been established as a risk factor for cardiovascular (CV) events independent of conventional CV risk factors [6–9]. Population-based studies in Western and Asian countries have shown that the risk of CVD increases as renal function declines. Because of this finding, the National Kidney Foundation formed a task force to heighten awareness of CVD in CKD, and defined CKD using parameters such as decreased eGFR < 60 ml/min/ 1.73 m^2^. A cohort of CKD patients closely observed by the physicians is required to accurately analyze renal and CV events. However, few studies have been conducted on the prevalence of LVH in a pre-dialysis population [10–13].

The aim of the present study was to clarify whether there is a close correlation between the prevalence of LVH and the stage of CKD classified according to eGFR and to identify factors related to LVH among the participants in the Chronic Kidney Disease Japan Cohort (CKD-JAC) [14].

## Subjects and methods

### Inclusion and exclusion criteria

Baseline characteristics of CKD-JAC are described elsewhere [15]. The following inclusion criteria were used at screening: (1) Japanese or Asian patients living in Japan; (2) age 20–75 years; and (3) a broad spectrum of CKD with eGFR of 10–59 ml/min/1.73 m^2^. eGFR was calculated using a modified three-variable equation for eGFR in Japanese patients [16]: eGFR = 194 × age^− 0.287^ × sCr^− 1.094^ (× 0.739, if female), where sCr = serum creatinine.

All patients were classified on the basis of CKD stage as described in our previous paper [14]. The following patients were excluded from participation: (1) patients with polycystic kidney disease, human immunodeficiency virus (HIV) infection, liver cirrhosis, active cancer, and patients who had received cancer treatment within the past 2 years; (2) transplant recipients and patients who had previously been on long-term dialysis; (3) patients who refused to provide informed consent.

Information on past medical history, including hypertension, acute myocardial infarction, angina pectoris, congestive heart failure, peripheral arterial disease, cerebrovascular disease, and prescription of antihypertensive agents, including angiotensin-converting enzyme (ACE) inhibitors, angiotensin receptor blockers (ARBs), calcium channel blockers (CCBs), diuretics, and b-blockers, statins, and antiplatelet agents, was collected from the medical records at each institution.

### Blood pressure and echocardiographic measurements

Blood pressure (BP) was measured in outpatient clinics with an automated sphygmomanometer after a 5-min rest. BP in the right arm was measured three times at intervals of 1 min, and the mean values were used for analyses. A mercury sphygmomanometer was used to measure the BP of patients who had frequent premature contractions, atrial fibrillation, or atrial flutter. Pulse pressure was calculated by subtracting diastolic BP from systolic BP. A two-dimensional guided M-mode echocardiographic study was performed in 1178 patients at each institution. Of them, the cases whose left ventricular end-diastolic diameter was not measured nor body surface area could not be calculated were excluded. Finally, this study included 1088 cases. Measurements included the diastolic thickness of the interventricular septum (IVST) and left ventricular posterior wall (PWT), and the internal diameter of the left ventricle at the end of diastole (LVDd) and the end of systole (LVDs). The modified Penn cube formula was used to calculate LV mass [17]: 1.04 × [(0.1 × IVST) + (0.1 × PWT)] × 3 − [(0.1 × LVDd) × 3] × 0.8 + 0.6, and LV mass was adjusted for body surface area (LVMI). LVH was defined as LVMI > 125 g/m^2^ in men and > 110 g/m^2^ in women [18]. Relative wall thickness (RWT) was calculated at end diastole as 2PWTd/LVDd and considered to be increased if > 0.45. The LV geometry was categorized as follows: normal (no LVH and normal RW), eccentric hypertrophy (LVH and normal RWT), and concentric hypertrophy (LVH and increased RWT).

### Definitions of hypertension, diabetes, and dyslipidemia

Hypertension was defined as systolic BP ≥ 140 mmHg and/or diastolic BP ≥ 90 mmHg or taking an antihypertensive agent. Diabetes mellitus (DM) was defined as HbA1C ≥ 6.5% or taking an antidiabetic agent. Diabetic patients were identified as those with diabetic nephropathy as the primary cause of CKD. Dyslipidemia was defined as serum triglyceride level > 150 mg/dl, or serum high-density lipoprotein (HDL) cholesterol level < 40 mg/dl in men and < 50 mg/dl in women.

### Collection of biological samples and measurements

Whole blood, serum, and urine samples were collected for measurement of serum Cr, HbA1c, intact parathyroid hormone (iPTH), and urinary albumin and Cr levels at a central laboratory. Urinary albumin excretion was expressed as the albumin to Cr ratio (ACR). HbA1c was measured by the JDS method, and the value was converted to the A1C value measured by the NGSP method by adding 0.4% as determined by the Japanese Diabetes Society. Each clinical center measured serum Cr at each visit. A 24-h urine specimen was collected from each patient once a year to measure the amount of proteinuria.

### Statistical analysis

All variables are reported as mean ± SD and frequency. Descriptive statistics of baseline characteristics were calculated by CKD stage, sex, and the presence or absence of LVH. CKD stages were defined according to the patient’s eGFR. Chi-squared test and Student’s *t* test or one-way analysis of variance (ANOVA) were used to detect between-group differences. ACR values had a skewed distribution and were log-transformed to achieve a normal distribution. Logistic linear regression was used to investigate the relation of LVMI to eGFR, BMI, and log ACR. Univariate logistic regression analyses were performed in an attempt to identify factors related to LVH. Multivariate logistic regression analyses were used to identify independent variables related to LVH. We considered some variables that had a *P* value < 0.10 in univariate logistic regression analyses as independent variables for multivariate logistic regression analyses. The model included the variables as follows: sex, smoking status, complications of DM, dyslipidemia and hypertension, past history of congestive heart failure (CHF), angina pectoris, myocardial infarction (MI), stroke and peripheral artery disease (PAD), systolic and diastolic BPs, pulse pressure, BMI, eGFR, uric acid, ACR, A1C, iPTH, HDL cholesterol, triglyceride, calcium, phosphorus, and prescription of antihypertensive agents. The two-sided 95% confidence interval (CI) and odds ratio (OR) were calculated by estimation. A two-sided probability level of 5% was considered significant. All statistical analyses were performed using the SAS software program for Windows ver. 9.4 (SAS Inc. Japan, Tokyo, Japan).

## Results

### Baseline demographics and clinical characteristics of participants according to eGFR level

We studied 2966 participants. Eleven participants (1 liver cirrhosis, 8 cancers, and 2 others) were excluded from 2977 of baseline participants [13]. The baseline characteristics of the 2966 participants in the CKD-JAC study have been described previously [14]. Of them, the subjects in this study, i.e., those (*N* = 1088) who were examined by echocardiography (UCG), consisted of 694 Japanese men (63.8%) and 394 Japanese women (36.2%), 454 (41.7%) and 807 (74.2%) of whom had DM and dyslipidemia, respectively. Most of the subjects had hypertension (88.7%) and were being treated with an antihypertensive agent (92.5%), most of them with ACE inhibitors (25.8%)/ARBs (75.9%), as shown in Table 1.

**Table 1:** Baseline characteristics of the study population by eGFR

Variable	All patients	eGFR (ml/min/1.73 m2)				*P* Value
		Stage 3a ≥ 45	Stage 3b 30 to < 45	Stage 4 15 to < 30	Stage 5 < 15	
*N*	1088	128	349	427	184	
Age (years)	61.8 ± 11.1	56.4 ± 12.9	61.6 ± 11.2	62.7 ± 10.6	63.7 ± 9.4	< 0.0001
Sex [*n* (%)]						
Male	694 (63.8)	82 (64.1)	228 (65.3)	273 (63.9)	111 (60.3)	0.724
Medical history [*n* (%)]						
Hypertension	965 (88.7)	105 (82.0)	298 (85.4)	397 (93.0)	165 (89.7)	0.0006
Diabetes	454 (41.7)	54 (42.2)	142 (40.7)	178 (41.7)	80 (43.5)	0.9405
Dyslipidemia	807 (74.2)	97 (75.8)	255 (73.1)	319 (74.7)	136 (73.9)	0.9262
Cardiovascular disease						
MI	71 (6.5)	7 (5.5)	20 (5.7)	29 (6.8)	15 (8.2)	0.6954
Angina	113 (10.4)	9 (7.0)	35 (10.0)	45 (10.5)	24 (13.0)	0.3912
Congestive heart failure	60 (5.5)	4 (3.1)	18 (5.2)	25 (5.9)	13 (7.1)	0.4877
PAD	40 (3.7)	3 (2.3)	9 (2.6)	18 (4.2)	10 (5.4)	0.2855
Stroke	134 (12.3)	17 (13.3)	43 (12.3)	49 (11.5)	25 (13.6)	0.8814
BMI (kg/m^2^)	23.6 ± 3.8	24.1 ± 3.3	23.7 ± 3.9	23.5 ± 3.8	23.4 ± 3.6	0.3595
Blood pressure (mmHg)						
Systolic	132.7 ± 18.2	130.4 ± 17.2	129.7 ± 17.7	133.7 ± 18.2	137.8 ± 18.3	< 0.0001
Diastolic	76.0 ± 12.0	76.0 ± 10.8	75.1 ± 11.8	76.3 ± 12.1	77.0 ± 12.7	0.2876
Pulse pressure (mmHg)	56.7 ± 14.0	54.4 ± 14.1	54.7 ± 13.5	57.4 ± 14.0	60.7 ± 13.8	< 0.0001
Creatinine (mg/dl)	2.17 ± 1.07	1.10 ± 0.18	1.43 ± 0.24	2.31 ± 0.53	4.02 ± 0.85	< 0.0001
eGFR (mL/min/1.73 m^2^)	28.7 ± 12.7	50.8 ± 5.3	37.3 ± 4.2	22.4 ± 4.3	11.9 ± 1.9	< 0.001
Uric acid (mg/dl)	7.24 ± 1.52	6.51 ± 1.41	7.013 ± 1.35	7.47 ± 1.56	7.65 ± 1.58	< 0.001
Urinary protein (g/day)	1.6 ± 2.2	0.8 ± 1.8	1.3 ± 2.1	1.7 ± 2.2	2.4 ± 2.2	< 0.0001
Urinary albumin (mg/g Cr)	1064.4 ± 1475.8	538.9 ± 967.6	881.2 ± 1615.2	1164.5 ± 1375.2	1544.8 ± 1556.8	< 0.0001
Total chol (mg/dl)	195.0 ± 43.8	199.3 ± 36.5	198.4 ± 48.0	195.0 ± 41.2	185.9 ± 44.7	0.0152
Non-HDL chol (mg/dl)	141.1 ± 42.2	141.5 ± 36.7	143.0 ± 45.8	142.0 ± 39.5	135.4 ± 44.6	0.3141
LDL chol (mg/dl)	111.4 ± 34.3	116.2 ± 28.5	113.1 ± 38.3	110.8 ± 32.2	106.3 ± 34.2	0.0814
HDL chol (mg/dl)	54.2 ± 18.3	58.1 ± 19.0	56.1 ± 19.1	53.0 ± 18.0	50.8 ± 16.2	0.0014
Triglyceride (mg/dl)	170.7 ± 117.2	165.9 ± 141.9	166.0 ± 111.3	177.0 ± 123.0	168.6 ± 94.2	0.5993
Calcium (mg/dl)	9.0 ± 0.56	9.3 ± 0.4	9.1 ± 0.5	9.0 ± 0.5	8.7 ± 0.6	< 0.0001
Phosphorus (mg/dl)	3.5 ± 0.7	3.3 ± 0.6	3.3 ± 0.6	3.6 ± 0.6	4.1 ± 0.8	< 0.0001
iPTH (pg/ml)	104.9 ± 82.8	54.8 ± 24.0	66.6 ± 35.2	106.6 ± 58.8	207.2 ± 121.0	< 0.0001
CRP (mg/dl)	0.3 ± 1.0	0.1 ± 0.2	0.2 ± 0.5	0.3 ± 0.8	0.4 ± 1.9	0.1071
A1C (%)	6.0 ± 1.0	6.0 ± 1.0	6.1 ± 1.1	5.9 ± 0.9	5.9 ± 0.8	0.0227
Hemoglobin (g/dl)	12.2 ± 1.9	13.3 ± 1.8	13.0 ± 1.80	11.7 ± 1.5	10.8 ± 1.4	< 0.0001
Medication [*n* (%)]						
Antihypertensive agent	1006 (92.5)	107 (83.6)	320 (91.7)	404 (94.6)	175 (95.1)	0.0002
ARB	826 (75.9)	93 (72.7)	257 (73.6)	337 (78.9)	139 (75.5)	0.2771
ACEI	281 (25.8)	24 (18.8)	100 (28.7)	126 (29.5)	31 (16.8)	0.0014
CCB	627 (57.6)	57 (44.5)	174 (49.9)	271 (63.5)	125 (67.9)	< 0.0001
β-Blocker	290 (26.7)	26 (20.3)	71 (20.3)	131 (30.7)	62 (33.7)	0.0005
Statin	468 (43.0)	64 (50.0)	151 (43.3)	177 (41.5)	76 (41.3)	0.3615
Diuretic	371 (34.1)	24 (18.8)	108 (30.9)	161 (37.7)	78 (42.4)	< 0.0001
Antiplatelet	280 (25.7)	23 (18.0)	94 (26.9)	107 (25.1)	56 (30.4)	0.0885

*MI* myocardial infarction, *PAD* peripheral artery disease, *BMI* body mass index, *Chol* cholesterol, *LDL* low-density lipoprotein, *HDL* high-density lipoprotein, *iPTH* intact parathyroid hormone, *CRP* C-reactive protein, *ARB* angiotensin receptor blocker, *ACEI* angiotensin-converting enzyme inhibitor, *CCB* calcium channel blocker

CKD was stage 3a in 128 patients (11.8%), stage 3b in 349 patients (32.1%), stage 4 in 427 patients (39.2%), and stage 5 in 184 patients (16.9%) (Table 1). The prevalence of CVD comorbidity tended to be inversely proportional to eGFR, but the correlation did not reach statistical significance. The groups with stage 4–5 CKD were older and had higher systolic BP and pulse pressure, a higher prevalence of hyperuricemia and anemia, and higher grades of proteinuria and albuminuria than the groups with stage 3a and 3b CKD, and serum levels of phosphorus and iPTH in stage 4 and 5 CKD patients were significantly higher than those in stage 3a and 3b CKD patients. The frequency of antihypertensive agents, including CCBs and diuretics, was gradually increased in accordance with the progression of CKD stage.

### Analysis by sex

Since the proportion of male subjects was 63.8% in the study population, sex may have affected the results of the present study. As shown in Table 2, female subjects were younger and had a lower prevalence of hypertension, DM, and past history of MI and stroke than male subjects. In addition, female subjects had lower BMI, lower serum levels of Cr and uric acid, and lower hemoglobin concentration than male subjects. However, there was no significant sex difference in eGFR. Female subjects had higher serum levels of lipids, including total cholesterol, non-HDL cholesterol, low-density lipoprotein (LDL) cholesterol, and HDL cholesterol, and lower serum triglyceride level. Lower percentages of female subjects were prescribed antihypertensive agents, including ARB, CCBs and β-blockers, statins and antiplatelet agents. As shown in Table 5, menopause was not significantly associated with LVMI by univariate logistic regression analyses.

**Table 2:** Baseline characteristics of the study population by sex

Variable	All patients	Sex		*P* Value
		Female	Male	
*N*	1088	394 (36.2)	694 (63.8)	< 0.0001
Age (years)	61.8 ± 11.1	60.9 ± 11.7	62.3 ± 10.7	0.0402
Medical history [*n* (%)]				
Hypertension	965 (88.7)	335 (85.0)	630 (90.8)	0.004
Diabetes	454 (41.7)	147 (37.3)	307 (44.2)	0.026
Dyslipidemia	807 (74.2)	297 (75.4)	510 (73.5)	0.4928
Cardiovascular disease				
MI	71 (6.5)	8 (2.0)	63 (9.1)	< 0.0001
Angina	113 (10.4)	25 (6.3)	88 (12.7)	0.001
Congestive heart failure	60 (5.5)	17 (4.3)	43 (6.2)	0.1914
PAD	40 (3.7)	9 (2.3)	31 (4.5)	0.066
Stroke	134 (12.3)	31 (7.9)	103 (14.8)	0.0008
BMI (kg/m^2^)	23.6 ± 3.8	23.1 ± 4.1	23.9 ± 3.5	0.0014
Blood pressure (mmHg)				
Systolic	132.7 ± 18.2	131.5 ± 18.9	133.4 ± 17.7	0.0834
Diastolic	76.0 ± 12.0	74.8 ± 12.0	76.7 ± 11.9	0.0119
Pulse pressure (mmHg)	56.7 ± 14.0	56.6 ± 14.4	56.8 ± 13.7	0.880
Creatinine (mg/dl)	2.17 ± 1.07	1.85 ± 0.88	2.36 ± 1.13	< 0.0001
eGFR (ml/min/1.73 m^2^)	28.7 ± 12.7	28.4 ± 13.0	28.9 ± 12.5	0.5329
Uric acid (mg/dl)	7.2 ± 1.5	6.9 ± 1.5	7.4 ± 1.5	< 0.001
Urinary protein (g/day)	1.6 ± 2.171	1.3 ± 2.0	1.7 ± 2.3	0.0874
Urinary albumin (mg/gCr)	1064.1 ± 1475.8	1008.9 ± 1552.0	1095.8 ± 1430.4	0.3583
Total chol (mg/dl)	195.0 ± 43.8	208.4 ± 45.7	187.3 ± 40.7	< 0.0001
Non-HDL chol (mg/dl)	141.1 ± 42.2	148.3 ± 44.8	137.1 ± 40.2	0.0001
LDL chol (mg/dl)	111.4 ± 34.3	118.7 ± 35.3	107.3 ± 33.1	< 0.0001
HDL chol (mg/dl)	54.2 ± 18.3	61.2 ± 19.2	50.3 ± 16.6	< 0.0001
Triglyceride (mg/dl)	170.7 ± 117.2	160.7 ± 107.3	176.4 ± 122.2	0.0414
Calcium (mg/dl)	9.03 ± 0.55	9.15 ± 0.54	8.96 ± 0.55	< 0.0001
Phosphorus (mg/dl)	3.5 ± 0.7	3.80 ± 0.6	3.4 ± 0.7	< 0.0001
iPTH (pg/ml)	104.9 ± 82.8	109.5 ± 85.7	102.3 ± 81.0	0.1717
CRP (mg/dl)	0.3 ± 1.0	0.2 ± 0.4	0.3 ± 1.2	0.1370
A1C (%)	6.0 ± 1.0	6.0 ± 1.0	6.0 ± 0.9	0.9776
Hemoglobin (g/dl)	12.1 ± 1.9	11.5 ± 1.6	12.51 ± 1.9	< 0.0001
Medication [*n* (%)]				
Antihypertensive agent	1006 (92.5)	351 (89.1)	655 (94.4)	0.0015
ARB	826 (75.9)	284 (72.1)	542 (78.1)	0.0257
ACEI	281 (25.8)	101 (25.6)	180 (25.9)	0.9129
CCB	627 (57.6)	206 (52.3)	421 (60.7)	0.0072
β-Blocker	290 (26.7)	90 (22.8)	200 (28.8)	0.0321
Statin	468 (43.0)	201 (51.0)	267 (38.5)	< 0.0001
Diuretic	371 (34.1)	131 (33.2)	240 (34.6)	0.6557
Antiplatelet	280 (25.7)	66 (16.8)	214 (30.8)	< 0.0001

### Comparison of the study population with and without LVH according to CKD stage and sex

**Fig. 1:**
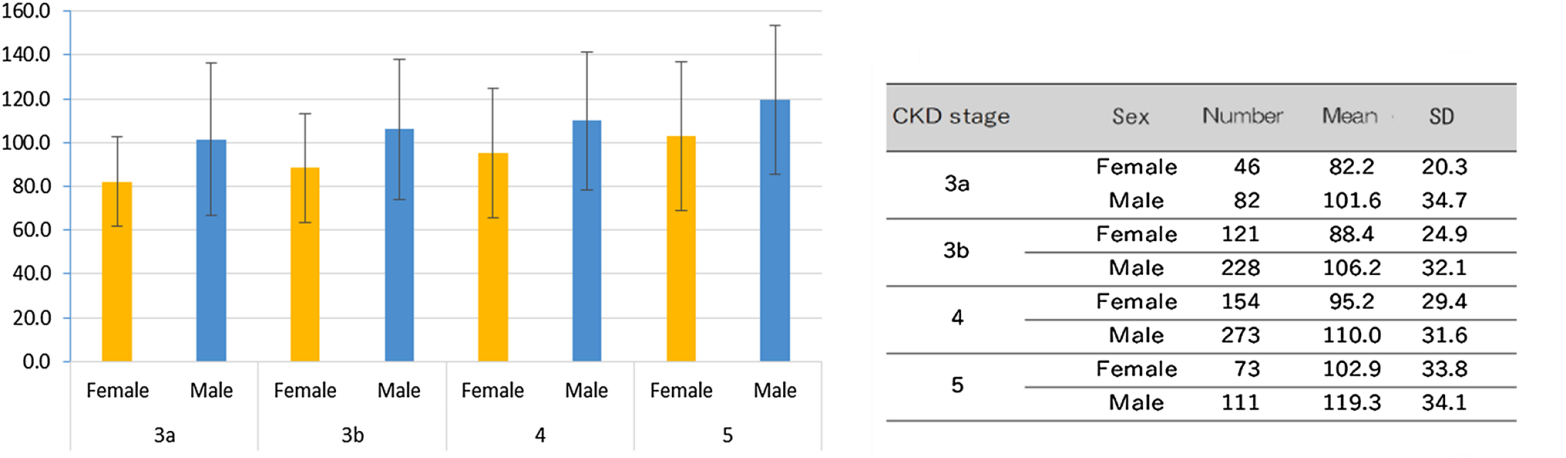
Relationship between estimated glomerular filtration rate (eGFR) and left ventricular mass index (LVMI) of patients with stage 3–5 CKD. **a** Female, **b** male

LVMI in each of the four groups of CKD patients according to eGFR is shown in Fig. 1 and tended to increase with the stage of CKD (*P* = 0.0005 in men, *P* = 0.0016 in women). The prevalence of eccentric and concentric LVH was higher among patients with more advanced CKD stages (G3a: 7.8 vs. 7.8%, G3b: 8.9 vs. 9.2%, G4: 10.8 vs. 14.3%, G5: 14.7% vs. 20.7%). The prevalence of LVH was 255 of 1088 (23.4%) of the study population (Table 3). Men had a higher prevalence of LVH than women (25.1 vs. 20.6%). The demographic and biochemical parameters of the study population are compared in Table 4. Female subjects with LVH had a higher prevalence of DM, past history of MI, and CHF and stroke, higher systolic BP, higher pulse pressure, lower eGFR, and higher ACR than female subjects without LVH. In addition, female subjects with LVH had lower serum HDL cholesterol level, lower calcium level, and higher serum levels of phosphorus and iPTH than female subjects without LVH. Moreover, female subjects with LVH had higher serum CRP value and lower hemoglobin concentration, when compared with female subjects without LVH. Finally, higher proportions of female subjects with LVH were being treated with CCBs, β-blockers, diuretics, and antiplatelet drugs.

**Table 3:** Baseline characteristics of the study population by LVH

Variable	All patients	LVH		*P* Value
		LVH (−)	LVH (+)	
*N*	1088	833	255	
Age (years)	61.8 ± 11.1	61.2 ± 11.3	63.8 ± 9.9	0.0007
Sex (male, %)	694 (63.8)	520 (62.4)	174 (68.2)	0.0912
Medical history [*n* (%)]				
Hypertension	965 (88.7)	727 (87.3)	238 (93.3)	0.0075
Diabetes	454 (41.7)	322 (38.7)	132 (51.8)	0.0002
Dyslipidemia	807 (74.2)	615 (73.8)	192 (75.3)	0.6401
Cardiovascular disease				
MI	71 (6.5)	39 (4.7)	32 (12.5)	< 0.0001
Angina	113 (10.4)	71 (8.5)	42 (16.5)	0.0003
Congestive heart failure	60 (5.5)	32 (3.8)	28 (11.0)	< 0.0001
PAD	40 (3.7)	28 (3.4)	12 (4.7)	0.3181
Stroke	134 (12.3)	90 (10.8)	44 (17.3)	0.0061
BMI (kg/m^2^)	23.6 ± 3.8	23.4 ± 3.8	24.4 ± 3.6	0.0002
Blood pressure (mmHg)				
Systolic	132.7 ± 18.2	131.4 ± 17.4	137.0 ± 19.9	< 0.0001
Diastolic	76.0 ± 12.0	76.1 ± 11.6	75.7 ± 13.2	0.6413
Pulse pressure (mmHg)	56.7 ± 14.0	55.3 ± 13.1	61.3 ± 15.8	< 0.0001
Creatinine (mg/dl)	2.2 ± 1.1	2.1 ± 1.0	2.5 ± 1.2	< 0.0001
eGFR (ml/min/1.73 m^2^)	28.7 ± 12.7	29.8 ± 12.6	25.2 ± 12.4	< 0.0001
Uric acid (mg/dl)	7.2 ± 1.5	7.2 ± 1.5	7.4 ± 1.5	0.0517
Urinary protein (mg/day)	1.6 ± 2.2	1.4 ± 2.1	2.1 ± 2.5	0.0108
Urinary albumin (mg/gCr)	1064.1 ± 1475.8	934.5 ± 1378.1	1490.0 ± 1693.5	< 0.0001
Total chol (mg/dl)	195.0 ± 43.8	196.8 ± 43.1	189.1 ± 45.6	0.0195
Non-HDL chol (mg/dl)	141.1 ± 42.2	141.7 ± 42.1	139.2 ± 42.7	0.4682
LDL chol (mg/dl)	111.4 ± 34.3	111.3 ± 34.1	110.6 ± 35.1	0.8984
HDL chol (mg/dl)	54.2 ± 18.3	55.5 ± 18.9	49.7 ± 15.6	< 0.0001
Triglyceride (mg/dl)	170.7 ± 117.2	170.6 ± 121.1	171.2 ± 103.5	0.9444
Calcium (mg/dl)	9.0 ± 0.6	9.1 ± 0.5	8.9 ± 0.6	< 0.0001
Phosphorus (mg/dl)	3.5 ± 0.7	3.5 ± 0.7	3.7 ± 0.7	0.0003
iPTH (pg/ml)	104.9 ± 82.8	98.7 ± 79.2	125.5 ± 90.9	< 0.0001
CRP (mg/dl)	0.3 ± 1.0	0.2 ± 0.5	0.4 ± 1.8	0.0052
A1C (%)	6.0 ± 1.0	6.0 ± 0.9	6.1 ± 1.0	0.2286
Hemoglobin (g/dl)	12.2 ± 1.9	12.3 ± 1.8	11.7 ± 2.1	< 0.0001
Medication [*n* (%)]				
Antihypertensive agent	1006 (92.5)	766 (92.0)	240 (94.1)	0.2527
ARB	826 (75.9)	635 (76.2)	191 (74.9)	0.6642
ACEI	281 (25.8)	209 (25.1)	72 (28.2)	0.3153
CCB	627 (57.6)	452 (54.3)	175 (68.6)	< 0.0001
β-Blocker	290 (26.7)	181 (21.7)	109 (42.7)	< 0.0001
Statin	468 (43.0)	359 (43.1)	109 (42.7)	0.9208
Diuretic	371 (34.1)	256 (30.7)	115 (45.1)	< 0.0001
Antiplatelet	280 (25.7)	171 (20.5)	109 (42.7)	< 0.0001

**Table 4:** Baseline characteristics of the study population by sex and LVH

Variable	Female		*P* value	Male		*P* value
	LVH (−) (−)	LVH (+)		LVH (−)	LVH (+)	
*N*	313	81		520	174	
Age (years)	60.0 ± 11.9	64.2 ± 10.1	0.004	61.8 ± 10.9	63.7 ± 9.8	0.0506
Medical history [*n* (%)]						
Hypertension	267 (85.3)	68 (84.0)	0.761	460 (88.5)	170 (97.7)	0.0003
Diabetes	107 (34.2)	40 (49.4)	0.0117	215 (41.3)	92 (52.9)	0.008
Dyslipidemia	234 (74.8)	63 (77.8)	0.5742	381 (73.3)	129 (74.1)	0.8222
Cardiovascular disease						
MI	4 (1.3)	4 (4.9)	0.0374	35 (6.7)	28 (16.1)	0.0002
Angina	17 (5.4)	8 (9.9)	0.1435	54 (10.4)	34 (19.5)	0.0017
Congestive heart failure	10 (3.2)	7 (8.6)	0.0315	22 (4.2)	21 (12.1)	0.0002
ASO	6 (1.9)	3 (3.7)	0.3374	22 (4.2)	9 (5.2)	0.6027
Stroke	16 (5.1)	15 (18.5)	< 0.0001	74 (14.2)	29 (16.7)	0.434
BMI (kg/m2)	23.0 ± 4.1	23.6 ± 4.0	0.2269	23.6 ± 3.5	24.7 ± 3.4	0.0002
Blood pressure (mmHg)						
Systolic	130.3 ± 18.3	136.0 ± 20.6	0.0155	132.1 ± 16.9	137.5 ± 19.7	0.0005
Diastolic	74.8 ± 11.6	74.9 ± 13.9	0.9353	76.9 ± 11.5	76.1 ± 12.9	0.4245
Pulse pressure (mmHg)	55.5 ± 14.0	60.9 ± 15.2	0.0028	55.2 ± 12.5	61.4 ± 16.1	< 0.001
Creatinine (mg/dl)	1.8 ± 0.8	2.2 ± 1.0	< 0.0001	2.3 ± 1.1	2.6 ± 1.2	0.0007
eGFR (ml/min/1.73 m2)	29.8 ± 13.0	22.9 ± 11.5	< 0.0001	29.8 ± 12.3	26.2 ± 12.7	0.001
Uric acid (mg/dl)	6.9 ± 156	7.0 ± 1.4	0.376	7.4 ± 1.5	7.6 ± 1.5	0.1369
Urinary protein (mg/day)	1.2 ± 2.0	1.7 ± 1.7	0.2266	1.5 ± 2.1	2.2 ± 2.7	0.0338
Urinary albumin (mg/gCr)	865.8 ± 1466.4	1559.3 ± 1748.6	0.0004	976.1 ± 1321.4	1457.1 ± 1671.0	0.0002
Total chol (mg/dl)	210.2 ± 43.9	201.5 ± 51.9	0.1405	188.7 ± 40.5	183.0 ± 41.0	0.1325
Non-HDL chol (mg/dl)	148.7 ± 44.7	146.9 ± 45.6	0.7778	137.5 ± 40.0	135.7 ± 40.9	0.6332
LDL chol (mg/dl)	118.5 ± 34.8	119.5 ± 37.6	0.8396	107.0 ± 33.0	108.0 ± 33.4	0.7398
HDL chol (mg/dl)	62.5 ± 19.4	55.6 ± 17.7	0.0082	51.3 ± 17.3	47.1 ± 13.8	0.0062
Triglyceride (mg/dl)	160.0 ± 113.4	163.1 ± 79.5	0.8263	176.9 ± 125.2	174.9 ± 112.9	0.86
Calcium (mg/dl)	9.2 ± 0.5	9.0 ± 0.6	0.0012	9.0 ± 0.5	8.8 ± 0.6	0.0001
Phosphorus (mg/dl)	3.8 ± 0.6	4.0 ± 0.7	0.0005	3.3 ± 0.7	3.5 ± 0.7	0.0038
iPTH (pg/ml)	102.7 ± 78.0	135.5 ± 106.9	0.0022	96.2 ± 79.9	120.6 ± 82.0	0.0007
CRP (mg/dl)	0.2 ± 0.4	0.3 ± 0.5	0.0122	0.3 ± 0.6	0.5 ± 2.2	0.0353
A1C (%)	6.0 ± 1.0	6.1 ± 0.9	0.2007	6.0 ± 0.9	6.0 ± 1.0	0.5995
Hemoglobin (g/dl)	11.7 ± 1.5	10.9 ± 1.7	< 0.0001	12.7 ± 1.8	12.1 ± 2.1	0.0006
Medication [*n* (%)]						
Antihypertensive agent	277 (88.5)	74 (91.4)	0.4619	489 (94.0)	166 (95.4)	0.4989
ARB	230 (73.5)	54 (66.7)	0.2229	405 (77.9)	137 (78.7)	0.8142
ACEI	76 (24.3)	25 (30.9)	0.2265	133(25.6)	47 (27.0)	0.7086
CCB	154 (49.2)	52 (64.2)	0.016	298 (57.3)	123 (70.7)	0.0018
β-Blocker	60 (19.2)	30 (37.0)	0.0006	121(23.3)	79 (45.4)	< 0.0001
Statin	159 (50.8)	42 (51.9)	0.8658	200 (38.5)	67 (38.5)	0.9917
Diuretic	92 (29.4)	39 (48.1)	0.0014	164 (31.5)	76 (43.7)	0.0036
Antiplatelet	38 (12.1)	28 (34.6)	< 0.0001	133 (25.6)	81(46.6)	< 0.00

**Fig. 2:**
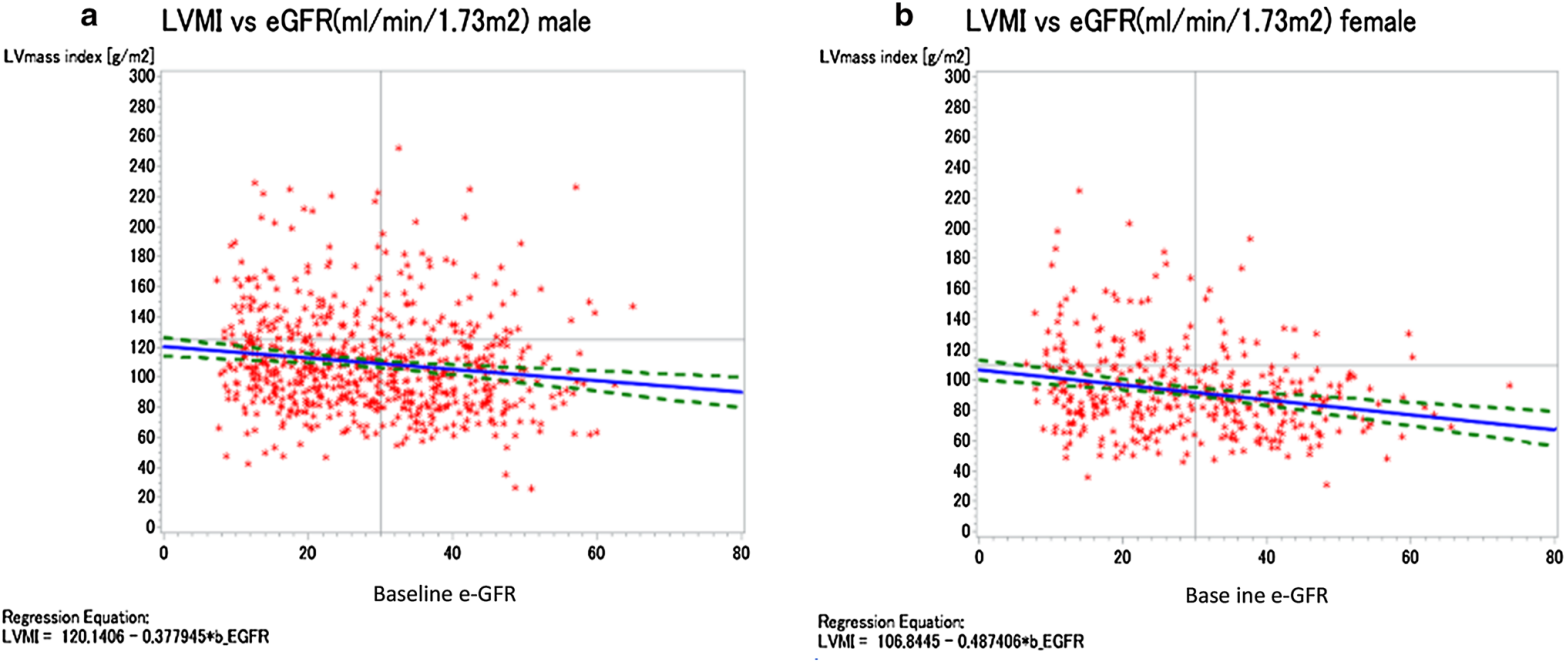
Relationship between body mass index (BMI) and left ventricular mass index (LVMI) of patients with stage 3–5 CKD. a Female; b male

**Fig. 3:**
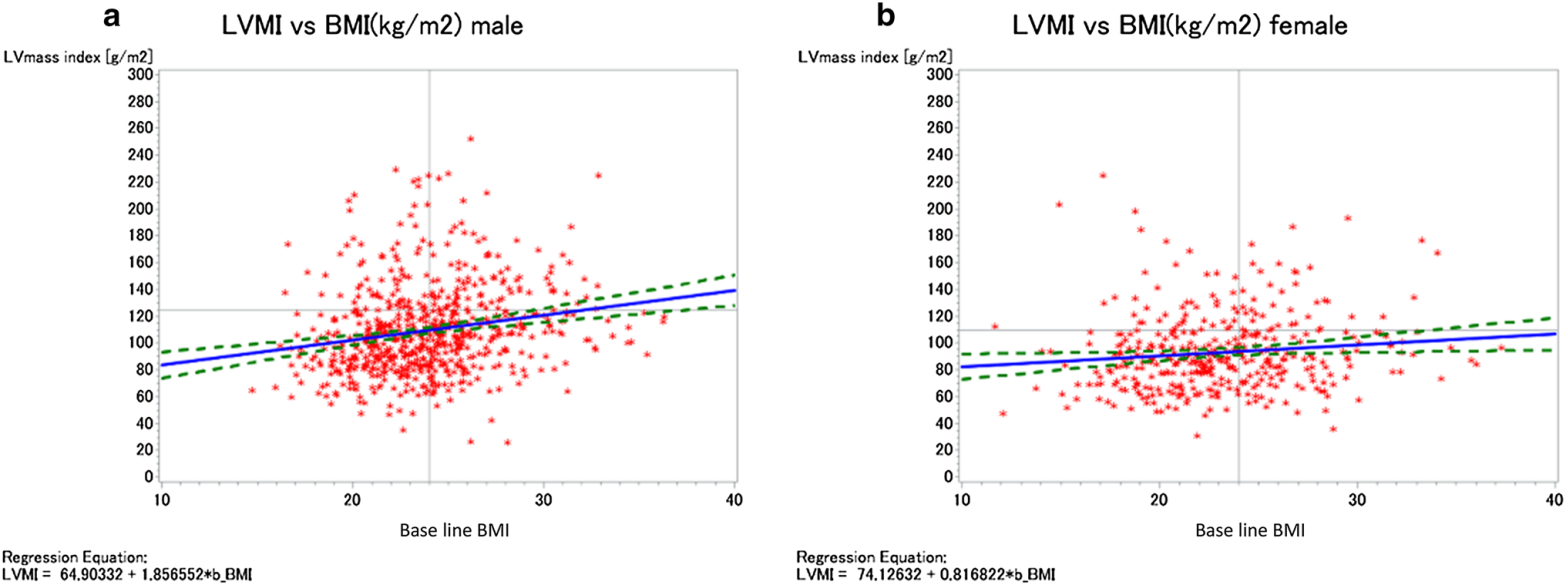
Comparison of left ventricular mass index (LVMI) in the different subgroups of CKD patients according to their degree of renal dysfunction

On the other hand, higher proportions of male subjects with LVH had hypertension and DM, and higher proportions had past history of MI, angina and CHF, when compared with male subjects without LVH. The group of male subjects with LVH had higher BMI, higher systolic BP, higher pulse pressure, lower eGFR, and higher ACR than female subjects without LVH. Among the lipid parameters, male subjects with LVH had significantly lower serum levels of HDL cholesterol level, when compared with male subjects without LVH. Parameters of mineral metabolism and hemoglobin concentration showed the same trends in female subjects as in male subjects with LVH. Moreover, higher proportions of male with LVH were being treated with antihypertensive agents similar to those in female subjects with LVH. LVMI was negatively associated with eGFR (Fig. 2), whereas LVMI was positively associated with BMI (Fig. 3).

### Factors related to LVH

Table 5 shows that the factors associated with LVH were age, DM, and hypertension. Past history of CVD except peripheral artery disease was significantly related to LVH. Significant clinical factors associated with LVH were systolic BP, pulse pressure, eGFR, BMI, ACR, serum levels of calcium, phosphorus, and iPTH, total cholesterol, HDL cholesterol, hemoglobin concentration, and prescription of erythropoiesis-stimulating agents and antiplatelet drugs.

**Table 5:** Factors associated with LVMI (univariate logistic regression analysis)

Variables	OR	95% CI	*P* value
Sex (female)	1.293	0.959–1.743	0.0917
Age (years)	1.024	1.010–1.039	0.0008
Smoking	1.06	0.707–1.589	0.7771
Menopause	1.327	0.906–1.944	0.1467
Complications			
Diabetes	1.703	1.284–2.259	0.0002
Dyslipidemia	1.08	0.781–1.493	0.6402
Hypertension	2.225	1.046–4.732	0.0377
Medical history			
Cardiovascular disease	2.477	1.848–3.320	< 0.0001
MI	2.921	1.789–4.771	< 0.0001
Angina	2.116	1.404–3.191	0.0003
Congestive heart failure	3.086	1.820–5.234	< 0.0001
ASO	1.42	0.711–2.835	0.3201
Stroke	1.722	1.164–2.547	0.0066
Blood pressure (mmHg)			
Systolic (10 mmHg)	1.182	1.094–1.277	< 0.0001
Diastolic (10 mmHg)	0.972	0.864–1.094	0.6409
Pulse pressure (mmHg)	1.03	1.020–1.040	< 0.0001
BMI (kg/m^2^)	1.703	1.034–1.113	0.0002
eGFR (ml/min/1.73 m^2^)	0.97	0.958–0.981	< 0.001
Uric acid (mg/dl)	1.096	0.999–1.202	0.0521
Urinary albumin (mg/gCr)	1.658	1.342–2.049	< 0.0001
A1C (%)	1.093	0.945–1.265	0.2291
Hemoglobin (g/dl)	0.835	0.771–0.905	< 0.0001
iPTH (pg/ml)	1.003	1.002–1.005	< 0.0001
Total chol (mg/dl)	0.996	0.992–0.999	0.0199
Non-HDL chol (mg/dl)	0.999	0.995–1.002	0.4678
LDL chol (mg/dl)	1	0.996–1.005	0.898
HDL chol (mg/dl)	0.98	0.971–0.990	< 0.0001
Triglyceride (mg/dl)	1	0.999–1.001	0.9441
Calcium (mg/dl)	0.505	0.387–0.658	< 0.001
Phosphorus (mg/dl)	1.456	1.182–1.792	0.0004
Medication			
Antihypertensive agent	1.399	0.785–2.495	0.2549
Statin	0.986	0.742–1.309	0.9209
ESA	1.497	1.014–2.209	0.0423
Phosphate binder	0.266	0.062–1.131	0.0729
Vitamin D	0.968	0.568–1.648	0.9035
Antiplatelet	2.89	2.142–3.900	< 0.0001

*OR* odds ratio, *CI* confidence interval, *ESA* erythropoiesis-stimulating agent

As shown in Table 6, the variables independently associated with LVH were past history of CVD, systolic blood pressure, BMI, urinary albumin, and serum total cholesterol level by multivariate logistic regression analysis.

**Table 6:** Factors associated with LVMI (multivariate logistic regression analysis)

Variables	OR	95% CI	*P* Value
Sex (female)	0.966	0.589–1.585	0.8926
Age (years)	1.017	0.994–1.041	0.1515
Smoking	1.021	0.593–1.76	0.9400
Hypertension	0.695	0.25–1.932	0.4857
Cardiovascular disease	2.364	1.463–3.822	0.0004
Stroke	1.063	0.581–1.945	0.8421
Systolic (10 mmHg)	1.173	1.005–1.369	0.0433
Diastolic (10 mmHg)	0.895	0.708–1.132	0.3544
BMI (kg/m^2^)	1.108	1.046–1.173	0.0005
eGFR (ml/min/1.73 m^2^)	0.992	0.971–1.015	0.5025
Uric acid (mg/dL)	1.024	0.898–1.168	0.7240
Urinaryalbumin(log mg/gCr)	1.425	1.028–1.974	0.0333
Total chol (mg/dL)	0.994	0.989–0.999	0.0174
HDL chol (mg/dL)	0.989	0.976–1.003	0.1375
Calcium (mg/dL)	0.83	0.549–1.255	0.3781
Phosphorus (mg/dL)	0.95	0.687–1.314	0.7561
A1C (%)	0.852	0.681–1.065	0.1602
Hemoglobin(g/dL)	0.955	0.827–1.103	0.5319
iPTH(pg/mL)	1.001	0.998–1.003	0.5423

*OR* odds ratio, *CI* confidence interval

As shown in Table 7, the variable independently associated with LVH in diabetic patients was only HDL cholesterol by multivariate logistic regression analysis. BMI has also some kind of relationship with LVH, but not significant. As shown in Table 8, the variables independently associated with LVH in non-diabetic CKD patients were past history of CVD, BMI, and total cholesterol by multivariate logistic regression analysis. Systolic BP and hemoglobin have also some kind of relationship with LVH, but not significant. Patients with eccentric and concentric LVH had significant risk factors with a history of previous CVD and higher BMI (Table 9).

**Table 7:** Factors associated with LVMI by diabetic CKD patients (multivariate logistic regression analysis)

Variables	OR	95% CI	*P* Value
Sex (female)	1.234	0.56–2.722	0.6022
Age (years)	1.014	0.979–1.051	0.4423
Smoking	0.85	0.401–1.802	0.6713
Hypertension	0.504	0.088–2.887	0.4420
Cardiovascular disease	1.5	0.761–2.956	0.2421
Stroke	1.036	0.459–2.339	0.9325
Systolic (10 mmHg)	1.132	0.909–1.41	0.2688
Diastolic (10 mmHg)	0.851	0.601–1.205	0.3633
BMI (kg/m^2^)	1.082	0.991–1.182	0.0789
eGFR (ml/min/1.73 m^2^)	0.986	0.956–1.018	0.3977
Uric acid (mg/ dL)	0.946	0.771–1.161	0.5967
Urinary albumin (log mg/gCr)	1.464	0.906–2.366	0.1195
Total chol(mg/ dL)	0.996	0.989–1.004	0.3196
HDL chol (mg/ dL)	0.971	0.947–0.996	0.0252
Calcium (mg/ dL)	0.753	0.431–1.315	0.3184
Phosphorus (mg/ dL)	1.192	0.746–1.903	0.4633
A1C (%)	0.84	0.633–1.115	0.2284
Hemoglobin(g/ dL)	1.095	0.891–1.344	0.3883
iPTH (pg/mL)	1.001	0.997–1.006	0.4784

*OR* odds ratio, *CI* confidence interval

**Table 8:** Factors associated with LVMI by non-diabetic CKD patients (multivariate logistic regression analysis)

Variables	OR	95% CI	*P* Value
Sex (female)	0.661	0.332–1.313	0.2367
Age (years)	1.029	0.995–1.064	0.0948
Smoking	1.082	0.464–2.523	0.8558
Hypertension	0.837	0.218–3.214	0.7958
Cardiovascular disease	3.968	1.915–8.219	0.0002
Stroke	1.05	0.41–2.691	0.9184
Systolic (10 mmHg)	1.254	0.98–1.604	0.0719
Diastolic (10 mmHg)	0.876	0.614–1.251	0.4669
BMI (kg/m^2^)	1.146	1.053–1.246	0.0015
eGFR (ml/min/1.73 m^2^)	1.008	0.975–1.041	0.6490
Uric acid (mg/dL)	1.086	0.906–1.302	0.3716
Urinary albumin (log mg/gCr)	1.372	0.843–2.233	0.2028
Total chol (mg/dL)	0.991	0.983–0.999	0.0244
HDL chol (mg/dL)	1.001	0.983–1.019	0.9148
Calcium (mg/d dL)	1.03	0.522–2.033	0.9317
Phosphorus (mg/dL)	0.704	0.432–1.148	0.1594
A1C (%)	0.632	0.293–1.363	0.2421
Hemoglobin(g/dL)	0.812	0.656–1.004	0.0547
iPTH (pg/mL)	1.001	0.998–1.005	0.4324

*OR* odds ratio, *CI* confidence interval

**Table 9:** Factors associated with eccentric and concentric LVH in CKD patients (multivariate logistic regression analysis)

Variables	Eccentric LVH OR	*P* value	Concentric LVH OR	*P* Value
Sex (male)	1.011	0.976	0.950	0.873
Age (years)	1.030	0.111	1.014	0.332
Smoking	0.696	0.430	1.171	0.628
Hypertension	1.577	0.667	0.417	0.138
Cardiovascular disease	2.386	0.011	2.235	0.008
Stroke	0.844	0.702	1.238	0.562
Systolic (10 mmHg)	1.199	0.114	1.141	0.176
Diastolic (10 mmHg)	0.825	0.271	0.960	0.784
BMI (kg/m^2^)	1.111	0.011	1.098	0.008
eGFR (ml/min/1.73 m^2^)	1.010	0.542	0.982	0.193
Uric acid (mg/dL)	0.929	0.448	1.098	0.264
Urinary albumin (log mg/gCr)	1.373	0.190	1.447	0.075
Total chol(mg/dL)	0.955	0.235	0.928	0.020
HDL chol (mg/dL)	0.952	0.617	0.857	0.097
Calcium (mg/dL)	0.823	0.566	0.882	0.618
Phosphorus (mg/dL)	0.857	0.527	1.043	0.837
A1C (%)	0.822	0.257	0.851	0.252
Hemoglobin(g/dL)	0.802	0.051	1.060	0.517
iPTH (pg/mL)	1.002	0.178	1.000	0.958

*OR* odds ratio, *CI* confidence interval

## Discussion

In the present cross-sectional study, we enrolled 2966 representative Japanese outpatients, most of whom had stage 3–5 CKD. These 2966 outpatients were being treated by nephrologists and were receiving a good standard of care. UCG was performed in 1088 of them. The UCG carried out was not intended to evaluate selected patients with cardiac complications, but was performed consecutively for evaluation of cardiac function in representative participants in the CKD-JAC study, if they provided informed consent. The prevalence (23.4%) of LVH in the present study was much lower than (30% <) that reported in previous studies in the pre-dialysis CKD population [19–21]. The participants in the CKD-JAC study may be better treated by nephrologists. Alternatively, cardiologists could treat more severe cases. The majority of the study subjects had hypertension and proteinuria or albuminuria on enrollment, but systolic and diastolic BP were prehypertensive (132/76 mmHg).

More than 90% of the subjects were being treated with antihypertensive agents (*n* = 1006, 92.5%), including ACE inhibitors (*n* = 281, 25.8%) and/or ARBs (*n* = 826, 75.9%).The prevalence rates of pre-existing CVD, i.e., MI (6.5%), angina (10.4%), CHF (5.5%), peripheral artery disease (3.7%), and stroke (12.3%), were higher than in the general Japanese population [22]. DM was present in 41.7% of the study subjects, and more than one-third of enrolled subjects had CKD secondary to glomerulonephritis. Subgroup analysis of LVMI and related factors of non-diabetic patients showed that history of previous CVD, BMI, and total cholesterol was significant and systolic BP and hemoglobin were almost significant (Table 8). However, those in diabetic patients showed that only HDL cholesterol was significant. Diabetes is considered to be a strong predictor for LVH, especially concentric LVH, in CKD patients as recently described [23, 24].

The results of the present study provided information on the prevalence of LVH and factors associated with LVH in stage 3–5 CKD patients in the CKD-JAC study. In the CKD-JAC study, LVH was observed in a small population (23.4%) of the 1088 study subjects, whereas LVMI tended to increase with the progression of CKD. In addition, the prevalence of eccentric and concentric LVH was higher among patients with more advanced CKD stages. CKD patients have a high prevalence of LVH, ranging from 34 to 74% in different studies, and its prevalence increases as renal function declines [11, 13, 25, 26]. However, the relatively wide heterogeneity of the prevalence of LVH in different studies can be attributed to several differences in the characteristics of the populations studied, including differences in ethnicity, age, proportion of subjects with different stages of CKD, prevalence of hypertension, method chosen to evaluate GFR, cutoff GFR used to enroll patients, and definition of LVH.

Elevated systolic BP has a continuous, graded, and independent association with risk of coronary heart disease, stroke, and ESKD [27]. LVH might be a beneficial compensatory process in CKD patients, allowing the left ventricle to produce additional force to increase cardiac work and maintain constant wall tension [28]. Even though mean systolic BP was well controlled (132.7 ± 18.2 mmHg), systolic BP was higher in patients with LVH than in patients without LVH in the present study. According to multivariate logistic regression analysis, systolic BP was an independent variable associated with LVH. Recently, it was reported that systolic arterial hypertension and elevated pulse pressure are closely associated with LVH in pre-dialysis patients, suggesting that fluid overload and increased arterial stiffness play important roles in LVH before starting dialysis therapy [13]. From the pathophysiologic standpoint, an increase in afterload induces concentric LVH, whereas volume overload leads to concentric LVH. In patients with CKD, the simultaneous coexistence of all factors (hypertension, arterial stiffness, volume expansion, and anemia) may preclude the development of specific alterations in LV geometry because of an overlap of different hemodynamic stimuli. Fluid volume management and maintenance of a near euvolemic state are crucial for the amelioration of LVH [29].

In accordance with the theory of non-hemodynamic LVH-promoting factors in our CKD patients, BMI was found to be a factor that was independently associated with LVH. Obesity is thought to be a risk factor independent of LVH, and heart disorders in obesity include structural adaptation with LVH and functional abnormalities [30]. Kotsis et al. [31] reported that obesity and daytime pulse pressure are predictors of LVH in true normotensive individuals. In hypertensive obese patients, metabolic syndrome (MetS) maintains its role as a risk factor for LVH independently of age and systolic BP and is a useful predictor of target organ damage in clinical practice [32]. However, MetS is no longer an independent risk factor when BMI is taken into account, suggesting that the effects of MetS on LVH are mainly driven by the degree of abdominal adiposity.

Currently, information about sex differences in renal abnormalities and CVD in healthy individuals is limited and conflicting. In the Prevention of Renal and Vascular End-Stage Disease (PREVEND) study, the prevalence of microalbuminuria in men was almost double that observed in women, and for a higher value of age and BMI was greater in men than in women [33]. In addition, the presence of CKD has been found to be associated with an increased risk of cardiovascular events [34] and of cardiovascular death [35] in both women and men having different degrees of cardiovascular risk or already having CVD. A recent study has shown that logistic regression analysis demonstrated that the factors significantly associated with the prevalence of LVH were age and BMI in women and uric acid in men [36]. In the present study, sex difference was not significantly associated with LVH in diabetic- and non-diabetic CKD patients. In our cohort, men had a higher prevalence of classical CV risk factors including hypertension, past history of previous CVD, hyperuricemia, and lower HDL cholesterol, suggesting that classical CV risk factors may be associated with LVH in men with non-diabetic CKD.

The results of the present study have shown that albuminuria is an independent predictor of LVH in CKD patients. A recent study reported that higher ACR was associated with LV mass, size, systolic function, and diastolic function in CKD patients [37]. This finding is in agreement with a previous international collaborative study showing a similar pattern for clinical risk of heart failure [38]. Even though the exact mechanisms behind the close link between albuminuria and altered LV structure and function are not clear, this may reflect the property of albuminuria as an indicator of systemic vascular damage, endothelial dysfunction, and microvascular injury in CKD patients [39].

Various abnormalities of mineral–bone metabolism are common in CKD patients, and mineral metabolism disorders such as hypocalcemia, hyperphosphatemia, and vitamin D deficiency have been found to be closely associated with CVD in CKD patients [40]. The mean serum calcium and phosphorus levels in the subjects of the present study were within the normal ranges, but differed between the groups with and without LVH. Serum iPTH level was elevated in patients with LVH and differed from that in the group without LVH. Hypocalcemia was associated with LVH by multivariate logistic regression analysis. Although its mechanism is not completely known, hypocalcemia followed by vitamin D deficiency may be associated with the pathogenesis of LVH. The results of the present study suggested that disorders of mineral metabolism may be involved in the etiology of LVH.

Some limitations have to be acknowledged in this study. First, selection bias cannot be ruled out, because patients were mostly enrolled at large-sized hospitals that can provide nephrology care [41]. Therefore, patients with greater awareness of their treatment probably were selected. Hence, the results from this study might not be applicable to the CKD patients who do not undergo appropriate treatment, are not managed at medical institutions, or do not realize that they have CKD because of the lack of prior medical examination [9]. Second, it is likely that there are possible biases of each study center in the performance of UCG. UCG was performed in most of the patients (80–90%) of each study center and tended to be underwent for high-risk patients. Thus, there are few selection biases for UCG performance in each study center. Third, because more than 90% of the patients were treated with ACR inhibitors and/or ARBs, serum potassium levels may be different between patients with and without these medications. Indeed, serum potassium levels (4.63 mEq/l) in patients with ACE inhibitors and/or ARBs were not significantly different from those (4.61 mEq/l) in patients with ACE inhibitors and/or ARBs (*P* = 0.55). Fourth, the definition of LVH in Japanese patients may be different from those in the USA. The LVMI value is dependent on body height and body weight. The cutoff values of LVMI were 125 g/m^2^ for men and 110 g/m^2^ for women in this study. These values were considered as 75 percentile of LVMI in this study. However, it is difficult to determine if the cutoff values of LVMI are suitable for use with the Japanese population. By contrast, the strengths of this study include its multicenter nature, the standard nephrology care, and the evaluation of patients with a wide range of eGFR.

In conclusion, the results of this study showed that the prevalence of LVH was low in stage 3–5 CKD patients treated by nephrologists in Japan. The cross-sectional baseline data from the CKD-JAC study shed light on the association between LVH and risk factors in patients with decreased renal function. Differences in the presence of previous CVD, blood pressure control, and metabolic state may lead to different outcomes of CVD in a longitudinal study. Future analysis of the CKD-JAC cohort will clarify whether the incidence of LVH varies with the causative disease during further follow-up.

## Appendix: Contributors

1. Steering Committee: Akira Hishida (Yaizu City Hospital), SeiichiMatsuo (Nagoya University), Tsuyoshi Watanabe (Fukushima Rosai Hosapital), Yasuo Ohashi (Chuo University), Hirofumi Makino (Okayama University), Tadao Akizawa (Showa University), Kosaku Nitta (Tokyo Women’s Medical University), Enyu Imai (Nakayamadera Imai Clinic)
2. Data Center: Public Health Research Foundation (Tokyo)
3. Independent Cardiac Function Evaluation Committee: Kyoichi Mizuno (Nippon Medical School Hospital), Hiroshi Nishimura (The University of Tokyo), Takeo Okada (Osaka Medical Center for Health Science and Promotion), Satoshi Iimuro (The University of Tokyo)
4. Biostatistics Adviser: Yasuo Ohashi (The University of Tokyo)
5. Medical Economics Adviser: Takashi Fukuda (The University of Tokyo)
6. Nutrition Evaluation Adviser: Satoshi Sasaki (The University of Tokyo)
7. International Adviser: Harold I Feldman (University of Pennsylvania)
8. General Adviser: Kiyoshi Kurokawa (National Graduate Institute for Policy Study).
9. Sponsor: Kyowa-Hakko-Kirin Co. Ltd.
